# Assessing the Effect of Incretin Hormones and Other Insulin Secretagogues on Pancreatic Beta-Cell Function: Review on Mathematical Modelling Approaches

**DOI:** 10.3390/biomedicines10051060

**Published:** 2022-05-03

**Authors:** Giovanni Pacini, Bo Ahrén, Christian Göbl, Andrea Tura

**Affiliations:** 1 Independent Researcher, 35142 Padova, Italy; giovannipacini49@gmail.com; 2 Department of Clinical Sciences, Lund University, 22184 Lund, Sweden; bo.ahren@med.lu.se; 3 Department of Obstetrics and Gynaecology, Medical University of Vienna, 1090 Vienna, Austria; christian.goebl@meduniwien.ac.at; 4 CNR Institute of Neuroscience, 35127 Padova, Italy

**Keywords:** insulin, secretagogues, beta cell, glucagon-like peptide-1, glucose-dependent insulinotropic polypeptide, glucagon, non-esterified fatty acids, amino acids, computational model, differential equation

## Abstract

Mathematical modelling in glucose metabolism has proven very useful for different reasons. Several models have allowed deeper understanding of the relevant physiological and pathophysiological aspects and promoted new experimental activity to reach increased knowledge of the biological and physiological systems of interest. Glucose metabolism modelling has also proven useful to identify the parameters with specific physiological meaning in single individuals, this being relevant for clinical applications in terms of precision diagnostics or therapy. Among those model-based physiological parameters, an important role resides in those for the assessment of different functional aspects of the pancreatic beta cell. This study focuses on the mathematical models of incretin hormones and other endogenous substances with known effects on insulin secretion and beta-cell function, mainly amino acids, non-esterified fatty acids, and glucagon. We found that there is a relatively large number of mathematical models for the effects on the beta cells of incretin hormones, both at the cellular/organ level or at the higher, whole-body level. In contrast, very few models were identified for the assessment of the effect of other insulin secretagogues. Given the opportunities offered by mathematical modelling, we believe that novel models in the investigated field are certainly advisable.

## 1. Introduction

In physiology, mathematical modelling has a long-standing tradition and has been focused on various aims [1,2,3,4,5]. A relevant aim has been the estimation of quantities that are not directly measurable or that would require invasive procedures to be measured, thus, not being feasible in the clinical routine. Another ambitious aim is using mathematical models as quantitative representations of specific physiological systems or processes, with the purpose of elucidating the mechanisms underlying the experimental measures and observations. In the field of insulin secretion and beta-cell function, mathematical modelling may have direct implications for anti-diabetes therapy, especially in the consideration that, currently, several pharmacological agents are available that can stimulate insulin secretion, such as the dipeptidyl peptidase-4 (DPP-4) inhibitors, glucagon-like peptide-1 (GLP-1) receptor agonists and sulphonylureas.

One of the first examples of mathematical modelling in insulin secretion and beta-cell function was the model by Grodsky and Ličko [6]. The model described the complex insulin secretion patterns observed in different in vitro and in vivo studies, and provided hypotheses on the possible mechanisms determining the beta-cell response, particularly after glucose stimulation. Some of these pioneering models were also summarized in “early” review studies [7,8,9].

Since those pioneering mathematical models were published, several further models have been developed in the field of insulin secretion and beta-cell function, again summarized in some reviews [10,11,12,13,14,15]. Of note, these more recent reviews typically focused on specific aspects of mathematical modelling in the field. For instance, some reviews focused on studies at the cellular level, describing mechanisms leading to insulin exocytosis from the beta-cell [10,11]. These mechanisms include the Ca^2+^ signaling pathways, the role of adenosine triphosphate (ATP) as a messenger mediating K^+^ channel behavior, the glucose-driven increase in cyclic adenosine monophosphate (cAMP) content of the beta cell, and the role of type 1 (GLUT-1) and type 2 (GLUT-2) glucose transporters in promoting the closure of the ATP-sensitive K^+^ channels [10,11]. Another review focused on the models describing the size distribution of the pancreatic islets (thus also of beta cells) and how their size changes under specific physiological and pathological conditions, such as aging, pregnancy, obesity, and diabetes [12]. In addition, a commentary article emphasized the opportunity and potential benefits of modelling approaches at a multi-scale level, which means building direct communication between models at different scales (cellular, organ/tissue and whole-body levels) [13], which has been scarcely performed so far, as also outlined by another study [14]. Other more general review articles on glucose metabolism and homeostasis also included information about models of insulin secretion and beta-cell function, possibly at different scale levels, such as in a recent studies of ours [15].

For several years, the studies on insulin secretion and beta-cell function (and related mathematical modelling) have mainly focused on the triggering action of glucose, being the most important insulin secretagogue. However, it has progressively become clear that several other compounds act (or may act) as insulin secretagogues, with typical effects of augmenting the action of glucose. The gut incretin hormones (the already mentioned GLP-1, and the glucose-dependent insulinotropic polypeptide (GIP)) are likely to be the most relevant substances that are able to enhance the glucose-induced insulin secretion (typically named as the incretin effect [16]), but other substances have proven able to stimulate insulin secretion. Following discoveries in the potential of different insulin secretagogues, mathematical modelling has progressed accordingly. However, to our knowledge, no previous study reviewed the mathematical models focused on the effect on insulin secretion and beta-cell function of incretin hormones and other non-glucose secretagogues. This review study aims to describe the main features of the mathematical models that have focused on the ability of incretin hormones, and other paracrine and endocrine substances, to affect insulin secretion and beta-cell function, both independently from glucose and in combination with glucose. Following the description of the approach used for the identification of the relevant studies, we present the related mathematical modelling, and then some concluding remarks, notes and comments.

## 2. Methodology for the Identification of Articles Describing Mathematical Models of Insulin Secretagogues

**Figure 1:**
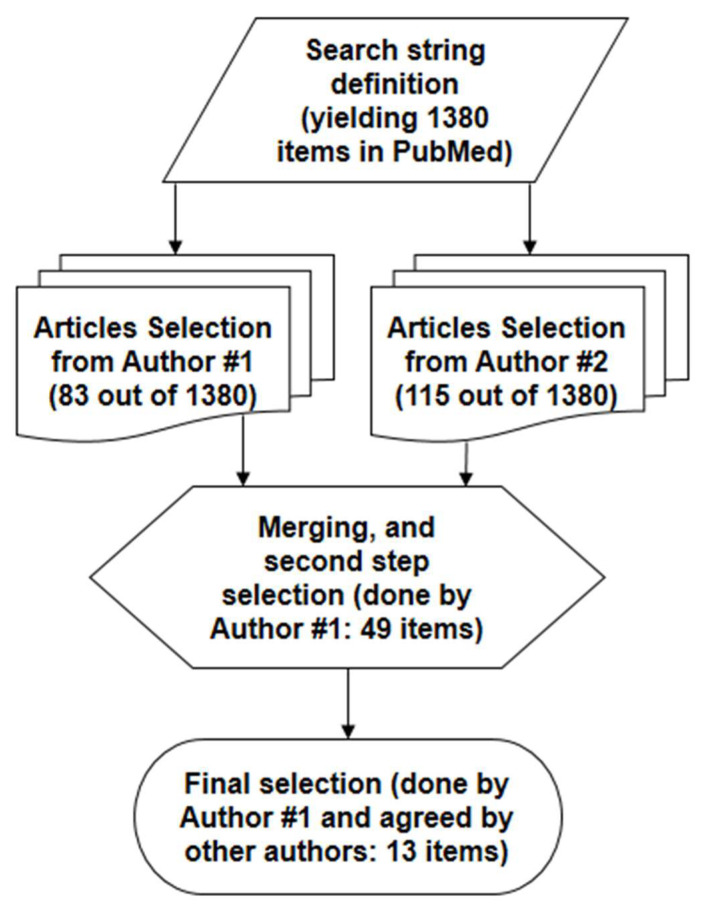
Flow diagram of the primary scientific literature search.

Our search of the scientific literature was performed in PubMed^®^. First, we searched for articles including the appropriate words, i.e., “model”, and one between “beta cell” or “insulin secretion” (or related variants). In addition, we requested the presence of at least one word (group of words) referring to insulin secretagogues, based on our *a priori* knowledge of the topic, that is, “incretin” (or specifically, “glucagon-like peptide-1” (“GLP-1”), or “glucose-dependent insulinotropic polypeptide”/“gastric inhibitory polypeptide” (“GIP”)), or “glucagon”, or “non-esterified fatty acid”, or “amino acid” (or, specifically, “arginine”) or, generically, “insulin secretagogue(s)”. Once agreed among the authors on the literature search string (providing 1380 items), the first step selection of the relevant articles was performed separately by two authors in December 2021, based on the inspection of the article title and possibly the abstract (both authors completed the relevant article selection by 17 December 2021). The authors then agreed on the final selection of the articles to be included in the analysis, possibly following the examination of the articles’ full text. This finally yielded a set of 13 articles, which were analyzed in detail and included in our review study. The details on the article selection procedure are reported in Figure 1.

In addition, based on the analysis of the reference list of the selected articles, we identified three additional articles that were missed by our literature search, thus, we used a total of 16 selected articles.

Furthermore, we considered a second search string based only on “model” and “beta cell” or “insulin secretion” terms, whose results were then restricted to “Review” or “Systematic Review” (yielding 297 items). We then selected some potentially interesting articles (25 in total), which were examined (especially their reference list) to possibly identify articles relevant for our review study that may have been missed by the primary literature search described above. However, none of those potential further articles were found to be relevant for our review; thus, at the end, none of the 25 articles were considered.

The specific details on the PubMed search strings are reported in the Appendix A.

## 3. Models of Incretin Hormones Effects on Insulin Secretion and Beta-Cell Function

Table 1 reports some basic information on the studies presenting mathematical models of the effects of the incretin hormones on insulin secretion and beta-cell function. Some details on each study are reported in the subsequent paragraphs.

One of the first two mathematical models, representing the effects of incretin hormones on insulin secretion, was published by Brubaker et al. in 2007 [17]. This model was built upon a 50-g and a 100-g oral glucose tolerance test (OGTT). It was based on a set of ordinary differential equations representing the changes in plasma glucose, insulin and the incretin hormones (GLP-1 and GIP), in addition to some equations simulating glucose entry into the system and changes in hepatic glucose balance. The incretin hormones kinetics were described by a single compartment model, with a distribution volume equal to 20% of the body weight. The equations included several nonlinear functions, in order to obtain a model with wide physiological and pathophysiological validity, although the effect of incretin hormones on insulin secretion was described by a simple linear additive term in the equation of plasma insulin time variation. The model was used to test oral glucose loads of different magnitudes, different rates of metabolic clearances, and several conditions, such as hypo and hyperglycemia, hyper and hypoinsulinemia, increases in insulin resistance (e.g., obesity) or, at converse, in insulin sensitivity (e.g., exercise training). It was claimed that all the glucose and insulin responses to these challenges were consistent with what had been reported in previous human studies. With regard specifically to the contribution of incretin hormones on insulin secretion, the simulation of a glucose load by preventing the oral glucose-induced rise in incretin hormones showed that, for the same size of the glucose load, the insulin response was reduced by 72%, as compared to what was observed with the traditional OGTT. Since it was reported that some studies indicated incretin hormones accounting for approximately 50–90% of the insulin response to oral glucose, it was concluded that the model reconstructed the incretin effect appropriately.

The other model, published in the same year as the previous one [17], was proposed by Jauslin et al. [18]. The model extended a previous model of the same research group and became adequate to study glucose and insulin levels in subjects with type 2 diabetes, following both an oral and an intravenous glucose administration (precisely, an isoglycemic glucose infusion, mimicking an oral glucose tolerance test profile). One peculiar aspect of the model was the description of the absorption phase of glucose, which was represented by a chain of transit compartments, through which the glucose dose was allowed to enter its central compartment, thus allowing appropriate modelling of the glucose absorption delay. The incretin effect was attributed mainly to GLP-1. Thus, to account for the GLP-1 effect, different types of relationships between the rate of glucose absorption and the secretion of insulin were hypothesized. Linear and nonlinear relationships were considered, either with a direct effect or with delay, and including both power and sigmoidal functions, to possibly maximize the effect of the absorption rate of glucose on insulin secretion. However, the details of these different incretin effect model formulations were not reported. It was, nevertheless, claimed that the model could be useful for simulating drug effects, reflected by the possible change in the value of some of the parameters when the model was exploited to analyze OGTT data following drug intake. This may yield deep insight into the mechanisms of action of new antidiabetic agents under examination. In addition, the model may help in decision making related to the development of the drug candidates, and in the definition of an optimized design of the clinical trials. Of note, a modified version of the model was presented a few years later for application to healthy subjects [19]. However, since we did not identify the relevant novelties in the incretin effect modelling, we do not report the specific description of this further study.

In 2010, Dalla Man et al. published a model of the GLP-1 effect on insulin secretion and beta-cell function [20]. To our knowledge, this is the first study including the effect of GLP-1 on a model that is able to dissect specific aspects of beta-cell function. Four models of the GLP-1 effect on insulin secretion were presented. All models shared the common assumption that insulin secretion includes two components (one proportional to the glucose rate of change and one to the absolute glucose values), but they differed in the modality of GLP-1 control. More precisely, the first model assumed a proportional effect of GLP-1 on suprabasal insulin secretion, whereas the second model assumed a proportional and a derivative GLP-1 effect; the third model assumed a nonlinear (Michaelis–Menten) GLP-1 effect, and the fourth model again added the derivative contribution to the nonlinear effect. The models were applied to the study of a group of healthy subjects that underwent a hyperglycemic clamp and two infusion rates of GLP-1. Glucose, GLP-1, and C-peptide data were used to determine the model that best represented the GLP-1 effect on insulin secretion (which was the one that provided the best fit of the measured C-peptide data). The optimal model was the one assuming that the suprabasal insulin secretion depends linearly on the GLP-1 concentration and its rate of change. The article suggested the need for further studies to assess the model validity during different metabolic protocols (e.g., meal test or OGTT), and its applicability to subjects with type 2 diabetes. Indeed, a modified version of the model was presented some years later for application to a mixed meal test, rather than the hyperglycemic clamp, thereby measuring GLP-1-induced potentiation of insulin secretion in response to a meal [21]. However, in line with our previous choice in a similar situation, since we did not identify the major novelties in the incretin effect modelling, we do not report the specific description of this further study.

The study by Takeda et al. in 2011 [22] presented a model of the effect of GLP-1 on the beta cell at cellular level. It was, therefore, different from the previous investigations [17,18,19,20,21] that presented models at the whole-body level. The model by Takeda et al. represented the GLP-1 receptor signal transduction in the beta cell, based on findings obtained from experiments carried out especially in beta cell and insulinoma cell lines. The model was fitted to the experimental data of the GLP-1 response, thus allowing the estimation of some unknown parameters describing the specific reaction steps. It was reported that the model satisfactorily reconstructed the dynamic changes in cAMP and predicted the action of cAMP effectors, protein kinase A, and cAMP-regulated guanine nucleotide exchange factors during GLP-1 stimulation. In addition, the model was able to predict the occurrence of two sequential desensitization steps of the GLP-1 receptor, which can be observed with both fast and slow reaction rates. The model was also able to reconstruct the cross talk between glucose and GLP-1-dependent signal cascades for cAMP synthesis. Of note, the model included several parameters; hence, sensitivity analyses were performed to assess how model output changed in relation to possible parameter variations. It should also be noted that this model was remarkably complex and also included partial differential equations (derivatives in space and in time) for the description of the diffusion of cAMP from the surface membrane toward the nuclear membrane. On one hand, this complexity indicates accuracy in the model description of the physiologic phenomena of interest, but on the other hand, it may discourage other investigators in using this model.

In the model presented in 2012 by Burattini and Morettini [23], the model by Brubaker et al. [17] was assumed as a basis to build up an integrated model applicable to a standard 75 g OGTT. Two different integrated models of the OGTT were obtained by incorporating two alternative representations of glucose absorption. The former incorporated a single compartment for the glucose absorption from the gut, and the derivative of a power exponential function for the gastric emptying rate, whereas in the latter, a nonlinear three compartment model was adopted to yield a more realistic multiphase gastric emptying rate. In both model versions, incretin hormone kinetics were described by a single compartment, and their effect on insulin secretion was described as an additive linear contribution in the equation of plasma insulin kinetics. A comparative analysis of the two model versions was performed in terms of their ability to reproduce the augmented glucose dependent plasma insulin concentration observed after the OGTT, as compared with the insulin response to an intravenous glucose infusion adjusted to match the OGTT glucose profile, which represented the incretin effect on insulin secretion. To this purpose, the mean data of OGTT and intravenous infusion tests (including both GLP-1 and GIP measurements) of metabolically healthy subjects were used to test the models’ capability in reproducing and interpreting the essential aspects of incretin-based insulin potentiation. A two-step procedure allowed the estimation of some adjustable parameters characterizing the gastric emptying rate, and the incretin, insulin and glucose kinetics. The two model versions proved substantial equivalence in matching the experimental data. The authors indicated the opportunity of model application for the assessment of the incretin effect in single individuals.

In 2013, De Gaetano et al. developed a model for the OGTT analysis [24], aimed at overcoming some limitations of previous models. Insulin secretion after glucose intake was represented by a mathematical formulation of the theoretically predicted insulin concentrations rather than by the experimental measures, which may be noisy. Furthermore, the glucose rate of appearance was derived by the absorption of glucose along the gastrointestinal tract, represented by a sequence of three compartments. This was assumed as the best compromise between accuracy and simplicity, compared to the excessively complicated approaches of some previous studies, where the glucose absorption was described as a continuous process. More precisely, one compartment was introduced to represent the stomach, whereas the three gastrointestinal compartments represented the jejunum, the ileum, and a delay between them (i.e., time needed for the food mass to travel from jejunum to ileum by peristalsis). Glucose entry into the gut caused the release of the incretin hormones, whose effect was assumed to stem from the glucose content in the jejunum and ileum. Specifically, the incretin effect was represented by a parameter describing a “glucose-concentration equivalent effect of incretins on insulin release”, depending on gut glucose content. It was claimed that incorporating in the model this incretin mechanism, and a limited progression of insulin release with increasing glycemia levels, were crucial in fitting the data, i.e., for the good performance of the model. However, it does not appear that the OGTT incretin concentration values were explicitly considered in the model, and this could be a limitation. On the other side, an interesting aspect of the model was that one of the estimated model parameters was found to have excellent correlation with known OGTT-based indices of insulin sensitivity.

**Figure 2:**
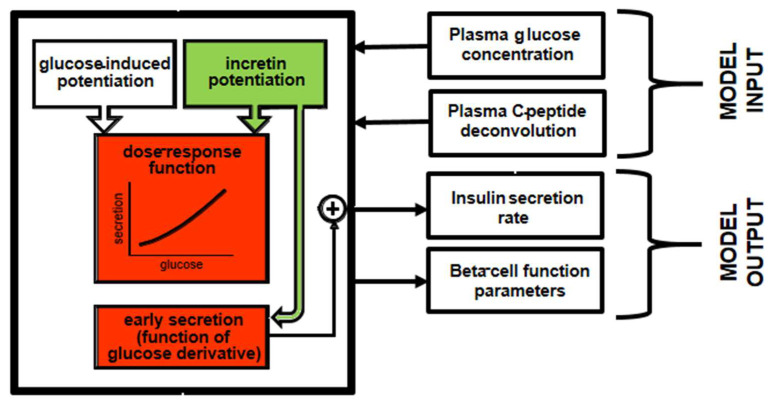
Schematic diagram of the model by Tura et al. [25] for the assessment of the incretin effect on insulin secretion and beta-cell function from an OGTT and an isoglycemic intravenous glucose infusion. The parts of the model related to the effects of incretin hormones are in a green color, whereas the parts affected by such effects are in red.

In 2014, Tura et al. presented a model for the assessment of the incretin effect, based on the analysis of an OGTT and related isoglycemic intravenous glucose infusion (IIGI) [25]. To our knowledge, this was—and still is—the only model able to perform contemporary analyses of both the glucose tests, thus resulting in more robustness compared to the models sequentially (separately) analyzing these tests. In this model, it is assumed that, during the IIGI, insulin secretion is attributable to different aspects of beta-cell function. These different aspects include the beta-cell glucose sensitivity (mean slope of the beta-cell dose-response), the glucose-induced potentiation of insulin secretion, representing the modulation of the dose-response due to glucose alone, and the rate sensitivity, quantifying early insulin secretion. During the OGTT, the incretin effect was modelled as the following two factors, which enhance the insulin secretion observed during the IIGT: an incretin-induced potentiation factor (that is, a further potentiation factor attributed, in fact, to the incretin hormones) acting on insulin secretion during the whole test duration, and a further increase in early insulin secretion. Figure 2 reports a schematic diagram of the model approach, with emphasis on the double effect attributed to the incretin hormones.

One of the advantages of the model was the opportunity to estimate the time course of the incretin-induced potentiation during the OGTT, and not simply an “overall” (average) parameter, as in other model approaches. It was observed that the incretin effect raises rapidly after glucose administration and remains sustained in normal glucose tolerance, it is transient in impaired glucose tolerance, and is virtually absent in diabetes. The model requires both OGTT and IIGI, but does not require incretin hormone data. Nonetheless, in datasets where plasma incretin data were available, it was found that the profiles of the model-estimated incretin effect were poorly related to those of the plasma incretin hormones, showing that such an effect is not necessarily related to the amount of incretin hormones released, and hence present in the systemic circulation.

An evolution of the model by Jauslin et al. [18] was published by Røge et al. in 2015 [26]. Jauslin’s model was modified for evaluating the effects of GLP-1 analogues on glucose homeostasis in patients with type 2 diabetes. The new model included the usual submodel for glucose and insulin homeostasis, with feedback mechanisms that control the insulin secretion and the elimination of glucose. To account for the effect of a GLP-1 analogue (liraglutide), a two-compartment model for liraglutide kinetics was coupled to the glucose-insulin submodel. In addition to the incretin effect due to the endogenous incretin hormones (as in Jauslin’s model), the liraglutide-induced incretin effect was considered. To describe this, a saturable function was exploited, linking the amount of ingested glucose (available in a glucose transit compartment) to the endogenous insulin secretion. The effect of liraglutide on glucose homeostasis was tested on different parts of the model (specifically, insulin secretion, endogenous glucose production, and glucose absorption rate). These model analyses showed that the action mode of liraglutide is mainly by the stimulation of insulin secretion following a glucose load, although a minor effect on gastric emptying may also be observed (depending on the type of nutrients included in the ingested meal). In contrast, no relevant effect was found for the endogenous glucose production. The model was claimed to be suitable for clinical trial simulations, based on the use of liraglutide, or other GLP-1 receptor agonists (possibly even combined), in the reasonable hypothesis of similar mechanisms of action.

In 2016, a new model was developed by Takeda et al. [27], following that published in 2011 [22]. This new model specifically aimed at explaining the molecular mechanisms and dynamic processes linking GLP-1-stimulated cAMP production to Ca^2+^ mobilization, as it was claimed that GLP-1 acts, at least partly, due to these cAMP-based mechanisms. Indeed, by GLP-1 action, cAMP facilitates the release of Ca^2+^ from the inositol trisphosphate receptor (IP_3_R)-regulated intracellular Ca^2+^ stores. The new model was constructed based on a previous model describing steady-state allosteric regulation of IP_3_R by Ca^2+^, as well as by the inositol trisphosphate itself. The model approach included one block aimed at modelling the steady-state open probability of the IP_3_R at different agonist concentrations and one block describing the dynamic properties of the IP_3_R, whose parameters were estimated to reproduce Ca^2+^ temporal variations generated in the beta cells. In addition, a “minimal” block was included, representing the minimum number of functional units for Ca^2+^ handling, consisting of multiple Ca^2+^ compartments that contained IP_3_R and some other pumps. It was claimed that the model was proven able to successfully reconstruct the Ca^2+^ transients and oscillations induced by GLP-1, as observed in beta cells from mice.

**Figure 3:**
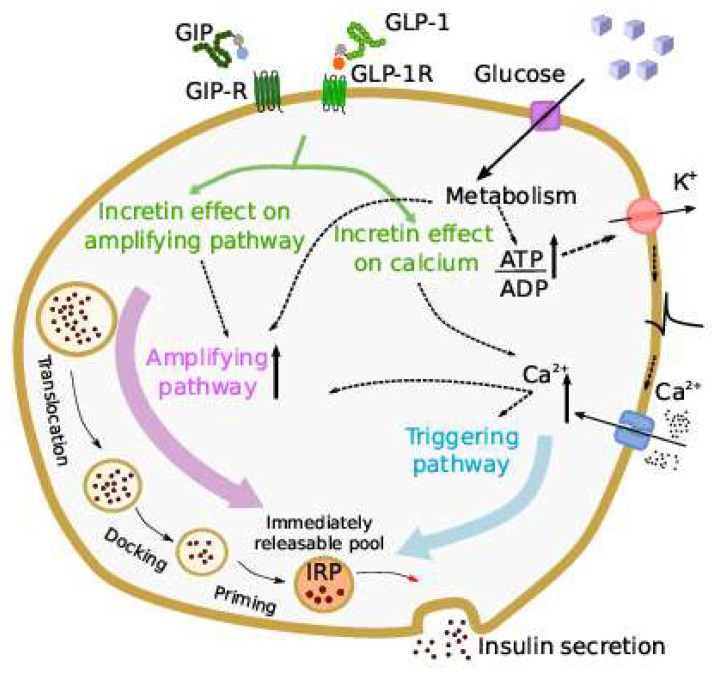
Scheme of the incretin effect in the model by Grespan et al. [28]. Incretins are assumed to act on both the triggering and amplifying pathways that regulate insulin secretion in beta-cells, increasing the cytosolic calcium (Ca^2+^) and amplifying insulin secretion through intracellular cAMP-dependent pathways (figure taken by Grespan et al. [28]).

In 2021, Grespan et al. [28] published a model investigating the different mechanisms of action of GLP-1 and GIP. The model was an extension of a previous model describing the different mechanisms of the insulin secretion response to intravenous glucose [29]. The new model, whose schematic representation is reported in Figure 3, included an immediately releasable insulin pool, the size of which depended on calcium-mediated exocytosis underlying the triggering pathway and the glucose- and calcium-mediated refilling flux, underlying the amplifying pathway.

Exocytosis was controlled by intracellular calcium, and insulin secretion was the product of a calcium-dependent function and the releasable pool size. On the other hand, the calcium level was determined from the glucose concentration by an appropriate submodel developed from mice data. To represent the effects of GLP-1 and GIP on insulin secretion, it was hypothesized that both incretin hormones may act on both the triggering and amplifying pathways. One peculiar and relevant aspect of the study was that the model was applied to several in vivo datasets, from studies employing either incretin hormones infusion or an OGTT. The main findings were that the stimulatory effects of GIP and GLP-1 differ, since the potentiation of insulin secretion increases linearly with GLP-1 over the whole dose range, while with GIP infusion, it reaches a plateau at about 100 pmol/L. In addition, the potentiation of insulin secretion by the two incretin hormones was found to be reduced in type 2 diabetes compared to nondiabetic individuals, but the entity of the reduction was somehow different between the two hormones (higher reduction with GIP). Furthermore, in our opinion, another interesting aspect of the study is that the proposed modelling approach can be considered as an example of multi-scale modelling (from cell to whole-body level).

## 4. Models of the Effects of Glucagon, Non-Esterified Fatty Acids, Amino Acids and Other Secretagogues on Insulin Secretion and Beta-Cell Function

Basic information on the studies that present mathematical models of the effects of glucagon, non-esterified fatty acids (NEFA), amino acids and other secretagogues on insulin secretion and beta-cell function are reported in Table 2.

With regard to glucagon, in the study published by Watts et al. in 2016 [30], the focus was not on insulin secretion and beta-cell function, but rather on glucagon secretion and alpha-cell function. The study started from the observation that glucose suppresses glucagon secretion either directly (through an intrinsic mechanism within the alpha cell) or indirectly through an extrinsic mechanism. Thus, a model was developed to describe the pancreatic islet that combined the models of pancreatic alpha, beta, and delta cells, with the main purpose of exploring the effects of insulin and somatostatin on glucagon secretion. It was found that the model was able to reproduce experimental data, demonstrating that the inhibitory effect of glucose remains even when paracrine modulators do not act on the alpha cell, and it was also demonstrated that paracrine interactions synchronize the alpha cell to produce pulsatile oscillations in glucagon secretion. On the other hand, the model also included the action of glucagon on the beta cell, and this is why this study was included in the present review. Indeed, it was noted that glucagon can increase insulin secretion by increasing cAMP levels, and this was modelled by an equation where glucagon increased the model parameter, governing the rate of production of the “priming” granules (“priming” being one of the states of the granules, in the cascade of steps leading to insulin granule exocytosis). It was also observed that the alpha cell can modulate insulin secretion by secreting acetylcholine, but this was not modelled explicitly. Since glucagon action on insulin secretion was not the focus of the study, no specific results were reported on this aspect of pancreatic cells interactions.

With regard to NEFA, in 2007 a model was proposed by Salinari et al. [31], in which insulin secretion rate was expressed as a function of both plasma glucose and NEFA concentrations. The model started from the observation that a rapid increase in fatty acids potentiates glucose-stimulated insulin secretion, by increasing the concentration of fatty acyl-coenzyme A (or complex lipids), which acts indirectly by modulating key enzymes (such as protein kinase C) or directly by modulating the exocytotic machinery. A simple linear first-order kinetic model was assumed to describe the relationship between plasma NEFA and NEFA within the beta cell, so that the temporal variations of the latter are a “smoothed” version of those of the former, with proportionality parameters representing the acyl-coenzyme A formation rate in cytosol from beta cell NEFA. Another model equation then described the nonlinear contribution of beta cell NEFA on insulin secretion in the presence of glucose. To evaluate the model performance in an experimental condition in which the incretin effect was minimized, data on insulin secretion following a lipid load and subsequent hyperglycemic clamp were analyzed. The model satisfactorily fitted the C-peptide concentration data of the hyperglycemic clamp, as well as data from the multiple-meal test over 24 h. It was concluded that the interaction between glucose and NEFA in the regulation of insulin secretion explains, at least partly, the potentiation factor of insulin secretion that is also observed following an intravenous infusion of glucose, when the incretin effect should be absent. Of note, the presented model can be considered another example of multi-scale modelling, describing phenomena within the beta cell and at whole-body level.

With regard to amino acids, one study was published in 2013 by Salvucci et al. [32]. The study was motivated by the fact that several amino acids induce insulin secretion by distinct phenomena, such as the depolarization of beta-cell plasma membrane either direct (e.g., by arginine) or induced by Na^+^ co-transport (e.g., by alanine), or by triggering and amplifying pathways linked to the Krebs cycle (e.g., by glutamine, leucine, alanine). The model focused, however, on the effects on insulin secretion specifically due to alanine. In fact, the model focused on two main alanine effects, i.e., the trigger of insulin secretion through the stimulation of oxidative metabolism, and the effect of membrane depolarization caused by alanine/Na^+^ co-transport on triggering Ca^2+^ influx. To this purpose, the following two submodels were developed: the first one described the core metabolic processes leading to ATP production with either glucose and/or alanine as input, whereas the second one described the electrophysiological downstream events, with metabolism-derived ATP as input and Ca^2+^ influx as output, eventually resulting in insulin granule exocytosis. The model was validated against the observations carried out on a functional clonal rat insulin-secreting beta cell line, in experimental procedures designed to obtain both single (glucose or alanine) and combined (glucose + alanine) acute stimulus dose-response curves.

With regard to other secretagogues, we did not find any article that explicitly focuses on endogenous secretagogues different from those previously reviewed. We only identified one somehow “pioneering” article, published in 1984 by Cohen and Pek [33], which was claimed to be applicable to insulin secretagogues in general, possibly in combination and/or in addition to glucose. The study moved from the notion that different phases and behaviors of insulin secretion can be observed under certain conditions. First, when the secretagogue reaches the beta cell, an acute phase of insulin secretion occurs within a few minutes. However, this phase dissipates rapidly, even in the case of continued exposure to the secretagogue. Then, the late phase of insulin secretion follows, which is maintained as long as the beta cell is exposed to the secretagogue. Under special conditions, upon the reduction in the secretagogue concentration, a third phase of secretion occurs. In fact, the secretion rate first returns to the basal level, then increases rapidly for several minutes, and finally dissipates in few minutes, with this phenomenon being named as the “off-response”. In addition, it is worth noting that the amount of insulin secreted during any of the phases may be affected by previous events (for instance, antecedent glucose administration can potentiate the beta-cell response to a second administration). The study by Cohen and Pek suggested that the metabolism of glucose is mandatory for the insulin off-response, which appears to occur in combination with the administration of leucine. The proposed model was able to describe all the phases of insulin secretion as determined by the stimulation of one or more secretagogues. The model also accounted for the indicated effect of antecedent exposure to secretagogues. From a mathematical point of view, the model was based on compartments with reciprocal influence. One compartment represented the exit of insulin secretory granules from the beta cell. Three other compartments were included to represent single metabolic events, or group of intimately related metabolic events. Four compartments allowed sufficient flexibility to reproduce a predominantly acute-phase response event, or a late-phase response, or an off-response. In fact, the model successfully reconstructed the insulin secretion experimental data derived by isolated perfused rat pancreas.

## 5. Summary of Studies, Concluding Considerations and Remarks

Our study reviewed the mathematical models that focused on the improvement/enhancement of insulin secretion and beta-cell function by the incretin hormones and other endogenous secretagogues. To the best of our knowledge, this is the first review study with this focus.

We found that the great majority of models relevant for the review addressed the role of the incretin hormones as insulin secretagogues, and this is on one hand reasonable, since incretin hormones are likely to be the most relevant insulin secretagogues, other than glucose. On the other side, it is undeniable that we were certainly expecting more studies presenting mathematical models that describe the action of other secretagogues. In contrast, as shown in Table 2, the number of these models, other than those “incretin hormones-related”, is modest, despite the fact that the potential insulinotropic activity has been determined for several substances (such as amino acids in particular, and partly NEFA and glucagon). This is particularly surprising in fact for glucagon, since several models have been developed even recently to assess glucagon secretion and/or glucagon kinetics [34,35]. Despite this, our review has shown that little attention has been devoted so far to the modelling of the effects of glucagon on the beta cell. Thus, our suggestion is that mathematical modelers in the field draw attention to the enhancing effect on insulin secretion of amino acids, NEFA and glucagon, whose action on the beta cell has been already established, at least under specific physiological conditions. This is, in fact, reported by several studies and also summarized by some reviews, such as that by Javed and Fairweather for the amino acids [36], that by Nolan et al. for NEFA [37], and that by Moede et al. for glucagon [38].

It is also worth noting that the majority of the models that we have identified in our study appears to be developed mainly for simulation purposes, rather than for parameter estimation in single individuals. The simulation of complex physiological systems, such as those involving insulin secretion and beta-cell function improvement by one or more secretagogues, is certainly relevant, since this can lead to better knowledge of the underlying physiological and pathophysiological mechanisms, possibly also suggesting new experimental investigations for further improving system comprehension. However, in our opinion, the role of models that allow the estimation of parameters from the clinical data of single subjects is similarly (if not more) relevant, especially when a precise physiological meaning can be attributed to such model parameters. In fact, when a model describes the physiology of the system or process under investigation (despite, of course, unavoidable assumptions and simplifications), the model parameters can assume specific physiological meaning, and this can lead to important clinical applications (often not immediately in the clinical routine, but certainly in several clinical trials). In particular, with regard to the type of models addressed in this review study, there are different potential clinical applications. Indeed, accurate assessment of the individual beta-cell function, including the degree of responsiveness to one or more secretagogues, may be important for a precise diagnosis of the beta-cell function defect. In addition, the assessment of individual sensitivity to the effect of a specific secretagogue may be important for individually tailored therapies. In fact, with regard to the effect of incretin hormones, several pharmaceutical agents are currently available (mainly the GLP-1 receptor agonists and the DPP-4 inhibitors), but other approaches may be suggested for individually defined strategies to potentially improve beta-cell function, such as appropriate nutritional prescriptions based, for instance, on supplementation with amino acids. These individually tailored approaches to the care of a metabolic disease appear as the incoming trend for the next years, as suggested in the recent recommendations concerning precision medicine in diabetes care [39,40].

It should be emphasized that the advantage of a clinically relevant model-derived parameter, compared to a non-model index with similar meaning, lies mainly in the robustness of the former, thus making it able to detect the possible subtle differences among different subjects’ populations, which may not be disclosed by the non-model indices. A clear example of this was observed in one of our previous studies in women with a history of gestational diabetes, which returned to normal glucose tolerance after pregnancy [41]. In that study, this group of women was compared to a group of women with no former gestational diabetes, matched by several physiological variables. When analyzing the beta-cell function of those two very similar groups, the non-model indices indicated similarity between them, whereas one model-based parameter (the previously mentioned beta-cell glucose sensitivity) was found slightly impaired in the former gestational diabetes group. The better ability in detecting subtle differences among groups for the model-based parameters, compared to non-model indices, likely lies in the above-mentioned increased robustness, which practically translates, for instance, to a lower tendency for being prone to outliers.

This study has some underlying hypotheses and limitations. It has to be noted that we only used PubMed to search for relevant articles. In principle, this is a limitation, but PubMed has wide journal coverage, including journals not strictly related to the biomedical area (such as bioengineering/biomedical engineering and even mathematics). In addition, our literature search strategy was wide in relation to the aims of the study, as mirrored by the large disparity between the number of articles returned from the primary search list, compared to the number of articles matching our review purposes, which were finally selected. Furthermore, we searched for relevant articles possibly missed by the search strategy by looking into the reference list of the selected articles, and in fact we identified some (although few) further articles to include. Thus, in our opinion, the hazard of having missed further relevant articles is small. However, it has to be acknowledged that we decided for some restrictions in our articles on inclusion criteria, as explained below.

In fact, we have to first mention that we excluded articles that were not written in the English language, and also excluded books/book chapters and Congress proceedings.

It is also worth noting that, in the field of beta-cell function, even simple indices may be useful, such as the widely used insulinogenic indices or its derivations [42]. However, we do not consider these indices as models. In fact, we name them as “empirical indices”, where “empirical” means that the index formula has been identified based on practical (empirical) considerations, typically without relevant physiological background. From a mathematical point of view, these indices are based on algebraic formulas. In our review, we focused instead on formulas based on differential equations, i.e., describing dynamic processes, and/or with relevant links to the system physiology or biology. To us, these are “models”.

In terms of models, some exclusions also have to be explained. First, our review focused on the endogenous insulin secretagogues. Thus, we did not include models of exogenous secretagogues (namely, pharmacological agents, such as the incretin analogues). Although such a choice may be questionable, we realized that a reasonably wide literature search to this purpose would have significantly enlarged the search that we have described. Thus, we agreed that such a focus may be an option for a future study. It is correct that, in one of the included articles, the model addressed the effect of a GLP-1 analogue [26], but this article was included in our review, since it also modelled the effect of endogenous incretin hormones (being, in fact, an evolution of a previous model included in our review [18]). Homology models were also not included. We identified some homology models with possible relevance to our review [43,44], but these types of models appear mainly lying in the framework of Computer Graphics, and hence very far from the mathematical models based on the differential equations that were of interest in this study. Moreover, it has to be mentioned that, in the field of diabetes and metabolism, some “general-purpose”, wide models have been developed, such as the Archimedes model [45], or the UVA/Padova simulator [46]. Due to their wide-encompassing nature, it cannot be excluded that they may have modelled the possible insulinotropic effects of substances other than glucose. However, we did not find explicit evidence of this and for this reason, we have not included them in our review. Finally, in this review, we were interested in the models of substances acting as insulin secretagogues; thus, the possible models of substances inhibiting, rather than enhancing, insulin secretion, such as somatostatin, diazoxide, ghrelin, and galanin, were excluded.

In conclusion, we reviewed the mathematical models dealing with the insulinotropic action of different endogenous substances. We found that a relatively large number of models has been proposed for the insulinotropic effect of the incretin hormones. In contrast, we identified a very small number of models focused on other compounds, despite their well-known insulinotropic action (as in the case of some amino acids). In the light of the advantages of mathematical modelling, leading to improved physiological knowledge, and to opportunities for individually tailored therapies and general care, we believe that the development of new models in the field is advisable and should be encouraged.

## Figures and Tables

**Table 1:** Basic information on the models of incretin hormone effects on insulin secretion and beta-cell function. In the “‘Tweet’ on model characteristic” field, a short description (≤200 characters) on the main model characteristics is reported. In the “Model aim classification” field, either “simulation” or “parameter estimation” is reported (on the individual subject’s data), based on what appears as the main application of the model; model application at cellular/organ level or whole body is also specified. To provide an indication of each study impact, the number of citations is reported in the “No. of citations” field (both the absolute number and the number per year, in square brackets). Source: Google Scholar (last checked: 31 January 2022).

Ref. No.	‘Tweet’ on Model Characteristics	Model Aim Classification	Use of In Vivo Human Data	Publication Year	No. of Citations
[17]	Model including a linear additive effect of incretins on plasma insulin, used to simulate hypo/hyper glycemia/insulinemia, and high/low insulin sensitivity conditions	Simulation (whole body)	No	2007	41 [2.7]
[18] ([19])	Model describing incretin effect as direct effect of glucose absorption rate (modelled as chain of transit compartments) on insulin secretion, used for simulating drug effects and clinical trial design	Simulation (whole body)	Yes	2007 (2010)	94 [6.3] (55 [4.6])
[20] ([21])	Model describing different aspects of beta-cell function, testing four possible effect types of GLP-1 on insulin secretion (linear, nonlinear, each plus possible derivative contribution)	Parameter estimation (whole body)	Yes	2010 (2016)	39 [3.3] (21 [1.8])
[22]	Model representing GLP-1 receptor signal transduction in the beta cell, able to reconstruct dynamic changes in cAMP and other factors at high GLP-1 levels (partial differential equations included)	Simulation (cell)	No	2011	23 [2.1]
[23]	Model including description of glucose absorption (two versions), with linear additive effect of incretins on plasma insulin, oriented to individual incretin effect estimation	Simulation (but oriented to parameter estimation; whole body)	Yes (but only average data)	2012	15 [1.5]
[24]	Model including particular representation of the gastrointestinal tract, with linear additive effect of incretins to enhance the glucose stimulus, also used for insulin sensitivity assessment	Parameter estimation (whole body)	Yes	2013	27 [3.0]
[25]	Model for concomitant analysis of OGTT and isoglycemic intravenous test able to provide several parameters of beta-cell function, used to assess incretin effect temporal profiles during the OGTT	Parameter estimation (whole body)	Yes	2014	42 [5.3]
[26]	Model for assessing the specific incretin effect after administration of a GLP-1 analogue, suitable for clinical trial simulations of one or even more GLP-1 analogues	Simulation (whole body)	Yes	2015	9 [1.3]
[27]	Model for explaining the molecular mechanisms and dynamic processes linking GLP-1-stimulated cAMP production to Ca^2+^ mobilization, able to reconstruct Ca^2+^ transients and oscillations induced by GLP-1	Simulation (cell)	No	2016	7 [1.2]
[28]	Model describing the differential effects of GLP-1 and GIP at beta-cell level, but applicable to whole-body data	Simulation (both cell and whole body)	Yes	2021	3 [3.0]

**Table 2:** Basic information on the models of the effect on insulin secretion and beta-cell function by glucagon, non-esterified fatty acids, amino acids, and other secretagogues. In the “‘Tweet’ on model characteristic” field, a short description (≤200 characters) on the main model characteristics is reported. In the “Model aim classification” field, either “simulation” or “parameter estimation” is reported (on the individual subject’s data), based on what appears as the main application of the model; model application at the cellular/organ level or whole body is also specified. To provide an indication of each study impact, the number of citations is reported in the “No. of citations” field (both the absolute number and the number per year, in square brackets). Source: Google Scholar (last checked: 31 January 2022).

Ref. No.	‘Tweet’ on Model Characteristics	Model Aim Classification	Use of In Vivo Human Data	Publication Year	No. of Citations
**Glucagon**					
[30]	Model mainly developed for assessing beta- and delta-cell actions on glucagon secretion, plus effect of alpha cell on insulin secretion	Simulation (cell)	No	2016	35 [5.8]
**Non-esterified fatty acids**					
[31]	Model describing contribution of non-esterified fatty acids to insulin secretion triggered by glucose, able to reconstruct data from hyperglycemic clamp and mixed meal tests in different populations	Parameter estimation (both cell and whole body)	Yes	2007	7 [0.5]
**Amino acids**					
[32]	Model describing the effects on insulin secretion of alanine through two distinct mechanisms, alone or in combination with glucose	Simulation (cell)	No	2013	24 [2.7]
**Other secretagogues**					
[33]	Model for reconstructing different phases of insulin secretion as triggered by possibly different secretagogues in combination and/or in addition to glucose	Simulation (cell)	No	1984	2 [0.1]

## Data Availability

Not applicable.

## Appendix A

This appendix reports some details on the search strategies of the scientific literature. The main PubMed search string was the following:

(model*[TI] AND (beta-cell*[TI] OR β-cell*[TI] OR (insulin[TI] AND secret*[TI])) AND ((amino*[TW] AND acid*[TW]) OR fatty acid*[TW] OR NEFA*[TW] OR FFA[TW] OR non-ester*[TW] OR incretin*[TW] OR glucagon-like*[TW] OR glucose-dependent*[TW] OR “gastric inhibitory”[TW] OR GLP-1[TW] OR GIP[TW] OR glucagon[TW] OR arginin*[TW] OR (insulin[TW] AND secretagog*[TW]))) OR (model*[TW] AND (beta-cell*[TW] OR β-cell*[TW] OR (insulin[TW] AND secret*[TW])) AND ((amino*[TI] AND acid*[TI]) OR fatty acid*[TI] OR NEFA*[TI] OR FFA[TI] OR non-ester*[TI] OR incretin*[TI] OR glucagon-like*[TI] OR glucose-dependent*[TI] OR “gastric inhibitory”[TI] OR GLP-1[TI] OR GIP[TI] OR glucagon[TI] OR arginin*[TI] OR (insulin[TI] AND secretagog*[TI])))

This search string was reached following several tentative strings. In our opinion, it allowed very high sensitivity in detecting the articles of interest (that is, missing of relevant articles highly improbable), with acceptable specificity (that is, limiting to a reasonable size the bunch of articles of no interest, which of course required some examination to reach a decision on discarding).

We requested that “model” and either “beta cell” or “insulin secretion” (or related variants) were terms (word expressions) present in the article title (TI PubMed search tag). In addition, we requested at least one specific term/expression, indicating consideration of an insulin secretagogue, present not necessarily in the article title, but essentially everywhere (TW PubMed search tag; see for details the following URL: https://www.ncbi.nlm.nih.gov/books/NBK3825/#pmchelp.Text_Words_TW (last checked: 26 January 2022)).

In addition, the search was completed with the complementary strategy. In fact, the string also searched for those articles with “model” and “beta cell” or “insulin secretion” everywhere (TW tag), and one insulin secretagogue in the title (TI tag).

We explicitly searched for those substances that are well known or even simply hypothesized (suggested) as insulin secretagogues. Of note, with regard to incretin hormones, we also searched individually for the glucagon-like peptide-1 (GLP-1) and the glucose-dependent insulinotropic polypeptide (GIP). With regard to the amino acids, we also searched explicitly for arginine. It is also worth noting that we searched generically for “insulin secretagogue” to further decrease the probability of missing any relevant articles.

We also considered the following additional search string:

model*[ti] AND (beta-cell*[TW] OR β-cell*[TW] OR (insulin[TW] AND secret*[TW]))

These string results were then limited, through the appropriate flags on the PubMed Web page, to “Review” and “Systematic Review”. With this search strategy, we searched for possible relevant review articles, whose analyses may have suggested further articles missed for any reason by the primary search strategy described above.
